# The Role of BMP Signaling in Female Reproductive System Development and Function

**DOI:** 10.3390/ijms222111927

**Published:** 2021-11-03

**Authors:** Esmeralda Magro-Lopez, María Ángeles Muñoz-Fernández

**Affiliations:** 1 Section Immunology, Laboratorio InmunoBiología Molecular, Hospital General Universitario Gregorio Marañón (HGUGM), 28007 Madrid, Spain; esmeraldamagrolopez@hotmail.com; 2 Instituto de Investigación Sanitaria Gregorio Marañón (IiSGM), 28007 Madrid, Spain; 3 Networking Research Center on Bioengineering, Biomaterials and Nanomedicine (CIBER-BBN), 28007 Madrid, Spain; 4 Spanish HIV-HGM BioBank, 28007 Madrid, Spain

**Keywords:** female reproductive system, embryonic development, BMPs family, BM signaling, signal transduction, human pluripotent stem cells, organoids

## Abstract

Bone morphogenetic proteins (BMPs) are a group of multifunctional growth factors that belong to the transforming growth factor-β (TGF-β) superfamily of proteins. Originally identified by their ability to induce bone formation, they are now known as essential signaling molecules that regulate the development and function of the female reproductive system (FRS). Several BMPs play key roles in aspects of reproductive system development. BMPs have also been described to be involved in the differentiation of human pluripotent stem cells (hPSCs) into reproductive system tissues or organoids. The role of BMPs in the reproductive system is still poorly understood and the use of FRS tissue or organoids generated from hPSCs would provide a powerful tool for the study of FRS development and the generation of new therapeutic perspectives for the treatment of FRS diseases. Therefore, the aim of this review is to summarize the current knowledge about BMP signaling in FRS development and function.

## 1. Introduction

The female reproductive system (FRS) is one of the most vital parts of the human reproductive process and is essential in keeping mammalian species alive. The FRS is responsible for the production of female germ cells (oocytes) and for the transport of oocytes to the fallopian tubes for fertilization and fetal growth and development [1].

**Figure 1:**
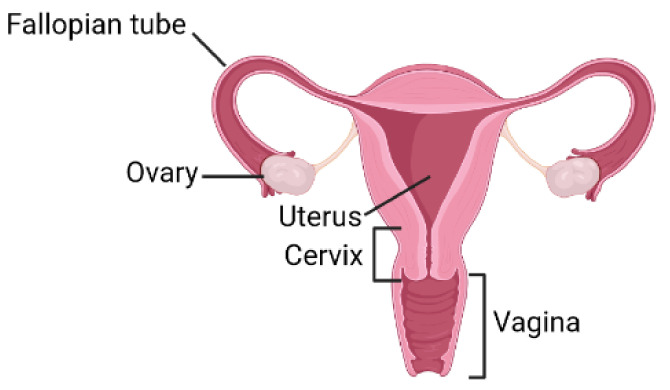
Anatomy of the female reproductive system (FRS). The FRS organs are the uterus, ovaries, fallopian tubes, cervix, and vagina.

The FRS is a collection of internal and external reproductive organs that work together with the aim of sexual reproduction and can be divided into three groups: gonads, reproductive ducts, and external genitalia [2]. The FRS consists of fallopian tubes, ovaries, uterus, cervix and vagina (Figure 1) [3].

Developmental anomalies in the formation and diseases of the FRS, including sexually transmitted diseases [4], cancer [5], or endometriosis [6], can result in infertility and life-threatening pregnancy or childbirth [1]. In spite of the importance of this system in the survival of the species, reproductive organs and the molecular mechanisms underlying their homeostasis, hormonal cycles, pregnancy, and associated diseases have been poorly characterized [7]. Despite the difference between animal and human FRS anatomy and molecular regulation, animal models have been very useful in understanding normal and abnormal FRS development, and so in predicting human FRS malformations [8].

The development of the FRS is a complex process that begins at an early stage in the embryo (at 5–6 weeks) [8]. Reproductive system development is closely related to the urinary system embryogenesis [1,9]. During embryogenesis, the development of the vertebrate urogenital system, which consists of the kidneys, gonads, and urinary and reproductive tracts, begins after gastrulation through the differentiation of the intermediate mesoderm (IM) [10]. The IM gives rise to two urogenital mesonephroi that compose the female (Müllerian) and male (Wolffian) primitive genital tracts [11]. The Wolffian ducts (WDs, also known as mesonephric ducts) differentiate into structures of the male reproductive tract, such as the epididymides, vas deferentia, and seminal vesicles [10]. Most of the FRS organs develop from the Müllerian Duct (MD, also known as the paramesonephric duct), whose development is preceded by WD growth. Following WD formation, the MD arises by the invagination of the anterior mesonephric coelomic epithelium (CE), and subsequently, the MD gives rise to the human FRS that further differentiates to form the human fallopian tubes, uterus, cervix and upper vaginal canal [2,12,13,14,15].

Several cellular and genetic/molecular mechanisms are involved in MD development and differentiation into the FRS organs. Although much progress has been made in identifying signaling pathways implicated throughout FRS development in recent years, there is still lack of knowledge of the developmental processes orchestrating FRS formation [13]. Several signaling pathways have been found to be involved in Müllerian/oviduct development, such as the bone morphogenetic proteins (BMPs), wingless-related integration site (WNT), transforming growth factor-β (TGF-β), the phosphatidylinositol 3-kinase (PI3K)/protein kinase B (Akt) pathway, the G-protein coupled receptor, and fibroblast growth factor (FGF) [7,8,9,13,14,15,16]. Among these signaling pathways, BMP signaling is essential in several processes during FRS development and function [17].

In this review article, we summarize the current knowledge about the role of BMP signaling in FRS development and function that might provide new clues for a better understanding of the contribution of BMP signaling in human FRS diseases and lead to the development of new therapeutic perspectives for their treatment.

## 2. The Family of Bone Morphogenetic Proteins (BMPs)

**Figure 2:**
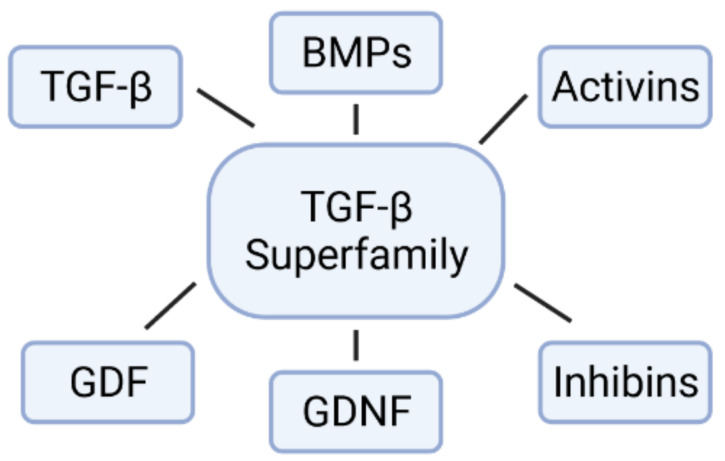
A schematic representation of TGF-β superfamily. GDF: growth and differentiation factor; GDNF: glial-derived neurotrophic factors.

The BMPs are multifunctional growth factors that belong to the TGF-β superfamily, which include TGF-βs, glial-derived neurotrophic factors (GDNFs), BMPs, growth differentiation factors (GDFs), activins, inhibins and others (Figure 2) [18]. The activity of BMPs was originally identified in the 1960s by their ability to induce ectopic bone formation [19]. These are important morphogens that play a key role during embryonic development and maintenance of adult tissue homeostasis. BMPs form a concentration gradient that activates differential genes that will give rise to distinct cell fates during dorsal–ventral (DV) axis patterning [20]. Different processes in early development are dependent on BMP signaling for the regulation of cell proliferation, differentiation, migration, organization, and apoptosis [18,21,22].

Although the name “BMP” might imply that the biological activity of all BMP members is to induce bone formation, some BMPs can act as inhibitors of bone formation, and other BMPs have been described to be critical in the development and maintenance of many organs and tissues (Table 1) [22,23].

**Figure 3:**
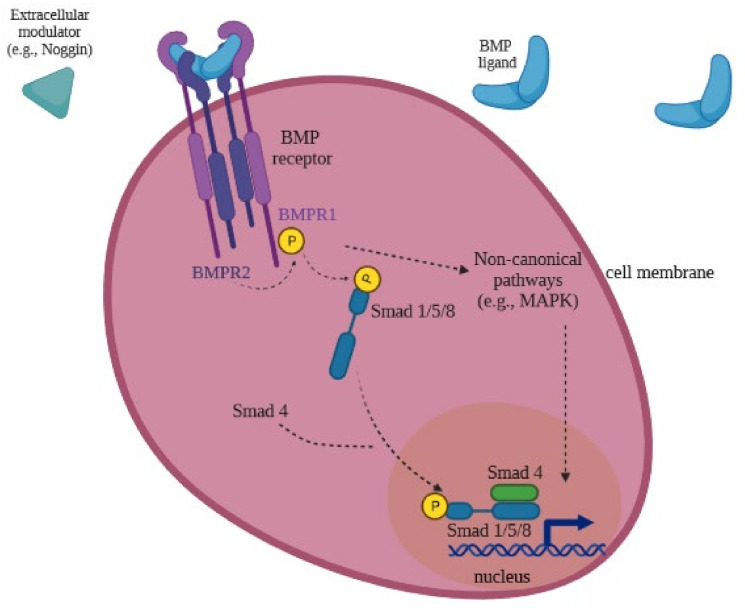
BMP cellular signaling mechanisms. BMP family members act by binding to type 1 and type 2 receptors (BMPR-1 and BMPR-2, respectively). Receptor activation leads to phosphorylation of Smad1/5/8 proteins by MPR-1 which complexes with Smad4 and translocates to the nucleus to bind Smad-binding domains of promoters in DNA to regulate gene expression [35,36,37].

Biological actions of BMPs are exerted through a hetero-oligomeric complex of the transmembrane serine/threonine kinase receptors type 1 and 2 (BMPR-1 and BMPR-2, respectively). Both receptors have a short extracellular domain, a single transmembrane domain, and an intracellular domain with serine/threonine kinase activity [18]. Receptors type 1 and 2 act as ligand-inducible transcription factors, inducing signal transduction pathways through SMADs. BMP ligands bind to the BMP receptors BMPR-1 and BMPR-2. BMPR-2 phosphorylates BMPR-1, and phosphorylated BMPR-1 phosphorylates particular members of the Smad family: Smad1, Smad5 and Smad8 (Smad1/5/8), which subsequently form a heteromeric complex with Smad4, translocate into the nucleus, and activate gene transcription in association with DNA-binding co-factors of various target genes [35,36,37] (Figure 3). BMPs might also transduce their signal through SMAD-independent pathways via mitogen-activated protein kinases (MAPKs), including extracellular signal-regulated kinases (Erks), c-Jun N-terminal kinases (JNKs) and p38 MAPKs [38]. The BMP signaling pathway mediates cell–cell communication during embryonic development and adult tissue homeostasis. In this sense, BMP signaling might be regulated on multiple levels: extracellularly, at the membrane site, and intracellularly. Extracellular regulators act as agonists or antagonists. Antagonists of BMPs, such as noggin and chordin, are secreted into the extracellular space, which bind to BMP ligands and block BMP signaling by preventing their association to the receptors [18,37]. The affinity of the BMP antagonists differs for BMP subtypes. For instance, noggin has an increased affinity for BMP-2 and BMP-4, and a low affinity for BMP-7 [39,40]. Intracellular regulators include microRNAs, phosphatases, inhibitory Smads (Smad 6 and 7), and methylation. Co-receptors in the membrane include endoglin, which seems to be important in vascular growth and disease [18].

## 3. BMPs Signaling in Female Reproductive System

BMPs are fundamental during embryogenesis, and a number of specific BMPs play an important role in aspects of reproductive system development and biology. Studies have reported findings such as gene knockout mice, as most BMPs are lethal before birth or shortly after birth [18]. In the following sections, we will discuss the advances in our understanding of the expression and functions of BMP members in reproductive cells and tissue, or BMPs in the FRS embryonic development. This would provide us with valuable information about the mechanisms underlying human development to generate 2D and 3D FRS organ models from hPSCs, a promising platform to understand biology and treat diseases.

### 3.1. BMPs in the Ovarian Function and Follicular Development

The ovarian follicle is the basic functional unit of the mammalian ovary. The follicle consists of the oocyte (germ cell) surrounded by somatic components (thecal and granulose cells) that are closely associated and interdependent [41]. BMP-1 is expressed in porcine ovarian follicle development and early embryogenesis, and it promotes oocyte maturation and has the ability to develop embryos during early in vitro culture [24].

BMP-2 is expressed in human cumulus cells (CCs) and shows a significant difference in the CCs of good quality oocytes, and so a good embryo [42]. BMP-2 is expressed in the granulosa cells (GCs) of atretic and graafian follicles in rat ovaries [43,44]. BMP-2 protein has been detected in the theca and oocytes of antral follicles in bovine ovaries [45]. BMP-2 treatment of human GCs showed effects in the production and expression of brain-derived neurotrophic factor (BDNF) via the upregulation of pro-BDNF and furin expression, which provides further insights in the modulation of follicular function in humans [46]. Moreover, BMP-2 was shown to promote human GCs and Gn-independent bovine (bGCs) proliferation via the upregulation of *SPHK1* mRNA expression through Hippo pathway suppression. These findings have important implications for directing treatment strategies for poor ovarian response (POR) in women with follicular dysgenesis [47].

*BMP-3* has been shown to be expressed in human GCs, showing a potential local regulator of female gonadal function. *BMP-3* mRNA levels were hormonally regulated by the human chorionic gonadotropin (hCG) in cultured human granulosa-luteal (GL) cells. Moreover, the activation of the protein kinase-A (PK-A) and protein kinase-C (PK-C)-mediated signaling pathways decreased BMP-3 mRNA levels in GL cells [26].

It is necessary to highlight the importance of BMP-4 signaling in folliculogenesis. BMP-4 protein expression has also been detected in all the stages of follicular development in the ovary of female mice. BMP-4 signaling is implicated in regulating oocyte and GCs [48,49] and has been demonstrated to significantly increase the number of in vitro derived-oocytes in a dose-dependent manner, a response which was inhibited in a dose–response relationship by co-treatment with noggin. However, the loss of BMP-4 functions leads to the loss of the primordial germ cell formation, the embryonic precursors of gametes (spermatocytes or ova) [50]. Moreover, BMP-4 was observed in the ovarian surface epithelium, in the corpus luteum and in the stromal cells around follicles [49,50,51].

Results have demonstrated that BMP-4 and -7 are strongly expressed in theca cells. Moreover, the effect of BMP-4 and -7 on GCs causes important changes in the follicle-stimulating hormone (FSH) action on estradiol and progesterone production in the mammalian ovary [52].

BMP-5 expression has been described in rat GCs as promoting specific effects on proliferation and steroidogenesis of GCs in an autocrine manner. These effects were shown to be associated with an increase in cyclin D2 protein level and a decrease in steroidogenic acute regulatory (StAR) protein expression in GCs in vitro. BMP-5 actions were inhibited by follistatin. These results showed that BMP-5 is a novel member of the BMP family that might play a fully paracrine role in rodent ovarian folliculogenesis [27]. BMP-6 was mainly expressed in oocytes at all human follicular developmental stages and *BMP-6* mRNA expression in CCs was not associated with oocyte maturation, embryo morphological grading, or implantation. *BMP-6* expression could therefore be used as a biomarker of oocyte maturation [28]. Treatment of human GCs with BMP-7 showed a functional link between clock gene expression and ovarian steroidogenesis [29].

After the arrival of germ cells into the gonads, gametogenesis begins. Molecular mechanisms underlying gametogenesis must be efficiently regulated to ensure the proper production of gametes. Members of BMP signaling control gametogenesis in a sex-specific manner. Several studies have shown a key regulatory role of the BMP signaling pathway in oogenesis in different species. For instance, BMP-6 and BMP-15 have been described to play a role in oogenesis [53]. *BMP-6* knockout female mice cause a decrease in fertilization success, with a lower number of ovulated eggs [54].

In a rat model, BMP-8 protein activates the SMAD1/5/8 and the SMAD2/3 pathways simultaneously in immature and mature GCs. At the same time, these two SMAD pathways also control the correct timing of folliculogenesis and luteinization [55]. The study has shown that the blockage of the SMAD1/5/8 pathway, but not the SMAD2/3 pathway, activated by BMP-8 can restore gonadotropin-induced progesterone production in GCs. This suggests that SMAD2/3 signaling activated by BMP-8 is not involved in the control of steroidogenesis [30].

BMP-15 plays a prominent role in ovarian function and female fertility and has been described specifically by increasing granulosa and thecal cells proliferation, differentiation, and function during follicular development. It has been shown to be essential for oocyte development, ovulation rate, fertilization, and embryonic competence [56]. The treatment of collared peccary preantral follicles with BMP-15 stimulated the activation of primordial follicles and maintained a high number of morphologically healthy follicles [57]. Some breeds, such as sheep, are carriers of important genetic variants in the ovulation rate. Naturally, gene mutations of the *BMP-15* ligand identified in sheep are associated with different ovarian phenotypic abnormalities. Sheep carrying heterozygous mutations in *BMP-15* (p.V299D/FecXI or p.Q291X/FecXH) have increased ovulation rates and litter sizes, whereas the homozygous state showed premature ovarian failure (POF) due to a blockage of folliculogenesis at the early stages [58]. Female *BMP-15* null mice showed decreased ovulation and fertilization rates [10,59].

Recent research has described the effects of BMPs (BMP-2, BMP-4, BMP-6, BMP-7, and BMP-15) on the regulation of *SERPINE2* expression through noncanonical SMAD2/3 and p38 MAPK signaling pathways. The results demonstrated that these BMPs induced differential upregulation of *SERPINE2* expression in primary and immortalized (hGL) cells. BMP-2 was shown to be the major modulator and has the best cellular activity. BMP-2 not only activated canonical SMAD1/5/8 signaling, but also noncanonical SMAD2/3 and p38 mitogen-activated protein kinase (MAPK) signaling [60].

### 3.2. BMP Receptors Signaling in the Female Reproductive Function

Numerous studies have investigated the cellular sites of expression of BMPs and BMP receptors in female reproductive organs, showing the importance of BMPs in female fertility. BMPR-1 and BMPR-2 receptors have been detected in uterine epithelia, in periluminal stroma and smooth muscle [11]. They also are implicated in regulating mammalian ovarian function and have shown expression in GCs and oocytes of follicles in the ovary [49].

A recent study has demonstrated that natural mutations in BMPR-1 receptors cause adverse effects on female fertility in humans. Genotyping of *BMPR-1A* and *BMPR-1B* genes in women affected by primary ovarian insufficiency (POI) facilitates the identification of novel variants. BMPR-1A-p.Arg442His and BMPR-1B-p.Phe272Leu variants have been shown to have a key role in the pathogenesis of POI. These findings reveal new molecular biomarkers for the diagnosis of POI in women [61].

Similar results have been found in *BmprIB*−/− female mice that show infertility due to estrus cyclicity, impaired pseudopregnancy responses, severe defects in CC expansion, and insufficient uterine endometrial gland development [62].

A *FecB^B^* mutation (g.A746G, p.Q249R) in the *BMPR-1* gene has also been identified to show increased ovulation rate and litter size in sheep breads. This gene influences follicular granulosa cell differentiation and follicular development, thus promoting ovulation [63,64]. A recent study has shown the potential use of gene editing technology to induce mutations in sheep (*FecB^B^* mutation), which would improve economically important traits in livestock [65]. The study of the effect of BMPR-1B gene modulation on granulosa cell function in goats resulted in altering their reproductive performance [66].

BMPR expression has also been shown during perinatal ovary development, especially during primordial follicle formation, and a positive relationship between FSH action and ovarian BMPR expression in hamsters has also been shown [67].

BMPR-2 is expressed in GCs during early folliculogenesis in ruminant primordial follicles and preantral follicles in rodents, and in all subsequent stages of follicular development [68]. Recent studies have demonstrated that the BMPR-2 receptor is required for gastrulation and mesoderm induction of mouse embryos [69]. Moreover, BMPR-2 has been described to be essential in uterine function after implantation for the development of the embryo after implantation and the maintenance of pregnancy in mice. BMPR-2 also regulates key pathways in uterine vascular development. The study showed that after normal implantation and early placental/fetal development, deletion of *BMPR-2* in the uterine deciduae of mice triggered midgestation abnormalities in decidualization that resulted in abnormal vascular development, trophoblast defects, and a deficiency of uterine natural killer cells. Results suggest that irregularities in uterine BMPR2-mediated signaling pathways might cause severe consequences for the maintenance of pregnancy in women. This finding allows us to use *BMPR-2* knockout mice models for studies on the diagnosis and treatment of these disorders [70].

### 3.3. BMPs Expression in Reproductive Tissue

Successful embryo implantation requires a competent blastocyst and a receptive endometrium [71]. Therefore, it is important to know the factors and signaling pathways that contribute to uterine receptivity for implantation.

BMP signaling plays an important role in the regulation of blastocyst implantation, endometrial stromal cell decidualization, and placental development [70,72,73].

BMP-2 is a key regulator of uterine function during implantation. A recent study demonstrates that female mice lacking BMP-2 in the uterus causes infertility due to abnormalities in uterine decidualization [44,74].

BMP-4 has been observed in the mouse uterus and has shown the strongest expression in the uterine luminal epithelium during the estrus phase [49].

A previous study has shown that BMP-5 null mice were viable and fertile [54]. However, the deletion of BMP-7 in the FRS causes female subfertility associated with defects during the early stages of pregnancy. Specifically, the absence of uterine BMP-7 results in a nonreceptive endometrium that causes improper implantation and additional defects throughout pregnancy [75].

A very recent report has shown that BMP-2 has a key role in the development of the placenta. The results show that BMP-2 promotes trophoblast differentiation in mouse trophoblast stem (TS) cells. Treatment with BMP-2 downregulated the expression of the TS cells and upregulated the expression of trophoblast giant cells (TGC). The effect of BMP-2 occurs at a very early stage of placentation [76].

BMP-4 is also an essential morphogen regulating vaginal development and is expressed in the early postnatal period [14]. A study has demonstrated that the BMP-4/ActA-SMAD/RUNX1 signaling pathway induces the vaginal epithelial cell fate in undifferentiated Müllerian duct epithelial cells (MDECs) by the activation of the ΔNp63 locus [77].

BMP-4 is expressed in adult vaginal submucosal smooth muscle cells. In the presence of estrogen, *BMP-4* expression is downregulated in vaginal smooth muscle. BMP-4 also mediates estrogen-driven plasticity of peripheral sensory innervation. Altered BMP-4 expression contributes to sensory hyperinnervation, a hallmark of several pain disorders, including vulvodynia. Estrogen appears to be a dominant factor driving reproductive tract neuroplasticity [7]. A previous report showed that estrogen suppressed BMP-4 synthesis in multiple mature tissue types and *BMP-4* expression increased in the prostate gland stromal cells of the estrogen receptor α knock-out mouse [12].

### 3.4. BMPs in the Embryonic Development of the Female Reproductive Tract

During embryogenesis, the mesoderm develops from the primitive streak (PS) and gives rise to CE. The MD emerges from the CE and differentiates into the FRS organs. BMP-4 represents the most potent ventralizing factor and has been shown to be essential in early development, beginning with embryonic gastrulation and mesoderm formation [48,78,79]. *BMP-4* knockout mice embryos have been described as dying at or about the time of gastrulation with no embryonic mesoderm development [48,79,80,81,82].

More recently, BMP signaling is known to regulate *Pax2* expression during IM development in chickens [61]. Previous experiments demonstrated that Lim1 is required for the maintenance and growth of MD cells in mice [83,84]. A recent study in mice has described that several BMP members, such as BMP-2, BMP-4 and BMP-7 have been shown to be involved in the MD forming region. Moreover, BMP signaling has been demonstrated to be required for *Pax2* expression in early MD development by adding the inhibitor of BMP signaling, noggin. Noggin caused a reduction of *Pax2* and *Lim1* signals and a disruption of MD invagination [85].

### 3.5. BMPs in the Differentiation of hPSCs into 2D Tissues and 3D Organoids

Human pluripotent stem cells (hPSCs) have unlimited self-renewal in vitro capacity and potential to differentiate into any cell type of the body, thereby offering the possibility of modeling human cells or tissues in 2D systems and organs in vitro in 3D models. Organoids are 3D in vitro tissue models that recapitulate many of the physiological key properties and features of the in vivo tissue. Both systems offer great promise for understanding basic mechanisms of human development, disease research, and potential for drug screening [67]. Numerous studies have featured the generation of organoids derived from primary tissues or hPSCs, such as the ovaries, fallopian tubes, endometrium, and cervix. Organoids can also be generated from disorders of the FRS such as endometriosis and cancer [86].

In recent studies, BMP-2, BMP-4 and BMP-7 have been demonstrated to play a role in the efficient differentiation of human embryonic stem (hES) cells in bipotential gonadal cells, although BMP-4 showed time-dependent effects [87]. Another important scientific contribution to reproductive biology is the generation of gametes, also known as in vitro gametogenesis, from mouse and human PSCs, which are an essential model for increasing our knowledge of human germ cell development and infertility. The differentiation process in mice needs the presence of BMP signals that are induced from the extra-embryonic ectoderm (ExE), such as BMP-4, BMP-2, BMP-8, and needed for the specification of primordial germ cells [62].

The efficient differentiation of hES cells into mesoderm has been a challenge for many years. BMP-4 ligand signaling has been identified as being required for mesoderm formation and many studies have shown that BMP-4 plays a central role in mesoderm induction and mesoderm lineage differentiation from hPSCs. Moreover, it has been demonstrated that BMP-4-deficient mice fail to develop mesoderm [88]. Recent research has described that short-term BMP-4 treatment (24 h) of hES cells differentiates into mesoderm at a high efficiency, and mesoderm progenitor cells are able to give rise to various mesodermal lineages. Short-term and long-term BMP-4 treatments on hES cell differentiation result in different effects, suggesting that the BMP signaling pathway might play a flexible and time-dependent role in human embryonic development and cell fate determination. While long-term treatment led to trophoblast and extra-embryonic endoderm differentiation, short-term treatment involved early mesoderm induction [89].

Recent research has described the directed differentiation of hPSCs into fallopian tube epithelium (FTE). The study showed Müllerian development by inducing IM differentiation, and further developed FTE precursors by modulating Wnt and BMP signaling [90].

## 4. Concluding Remarks and Future Directions

The general concept to arise from the studies described in this review is that BMP signaling is fundamental in embryogenesis and plays an essential role in FRS functions. For the past few years, significant progress has been made in the understanding of human FRS structure and function by revealing the roles of various BMP members in female reproduction. Cellular function and biological importance of BMP signaling in a wide range of embryonic and adult tissues are well known. Most BMPs play a key role in ovarian function and follicular development. They can also be involved in infertility, in improper implantation, or throughout pregnancy. BMPs might also modulate the effects of hormones on cell differentiation and proliferation. However, some functions of BMP members in the FRS still remain unknown, such as BMP-12, BMP-13, and BMP-14. In addition, the role of BMPs in vaginal tissue is also not well known. We also need to keep investigating new mutations in the BMP system that are involved in important physiological functions of BMPs in reproduction, or the molecular and cellular mechanisms of the BMP signaling pathways and examine the potential implications in different physiological and pathological conditions. However, we could use the advances already made and translate them into clinical and agricultural applications.

In spite of the findings described here in humans, most of the studies have focused on animals. The mouse has been used extensively as a model for conducting studies of human development and pathogenesis. Indeed, the cellular and molecular mechanisms implied in MD-derived organs have been extensively studied utilizing animal models. Although several reports have denoted remarkable similarities in molecular pathways during organogenesis of the FRS of humans and mice, some notable differences, such as anatomic, developmental, and endocrinologic differences, have also been described.

We are just beginning to understand the role of BMPs in the human reproductive system. In this sense, the successful differentiation of hPSCs provides a unique opportunity to model human organ development in a system similar to development in vivo. Organoids are becoming essential models that mimic in vivo human 3D organ structure and function, and have become a powerful tool in studying human development and in generating treatments for diseases of the FRS.

These human FRS models based on organoids can be used to introduce mutations and potentially mimic FRS human pathologies that have been associated with BMP family members. Currently, multiple methods of genome engineering (such as CRISPR-Cas9) have been employed in organoids for multiple purposes. This improves our understanding of the FRS molecular mechanisms underlying several diseases and may lead to developing new therapeutic drug candidates that may advance more quickly and effectively into clinical candidates.

Research with these models might also improve our knowledge enough to study the effect of combined BMP members in differentiation assays on the same cell or tissue type in humans or signaling cross-talk between TGFβ1/BMP and other pathways, such as WNT. It could also yield the necessary insights to generate new organoids of different tissue models that have not been generated yet. Finally, a better understanding of BMP signaling will facilitate the determination of the cause of the BMP signaling-associated human diseases and help to develop new therapeutic perspectives for their treatment.

## Figures and Tables

**Table 1:** Representative roles of the different BMP family members in the FRS development and function.

BMP Members	Function
BMP-1	BMP-1 has a role in ovarian follicular development and early porcine embryo development [24].
BMP-2	BMP-2 plays a key function in placenta development [25].
BMP-3	BMP-3 is expressed in human granulosa cells (GCs) [26].
BMP-4	BMP-4 is an essential morphogen regulating vaginal development and is expressed in the early postnatal period [14].
BMP-5	BMP-5 is expressed by GCs in the rat ovary and exerts biological effects on proliferation and steroidogenesis of GCs in an autocrine manner [27].
BMP-6	BMP-6 expression is negatively associated with oocyte maturation in cumulus cells (CCs) [28].
BMP-7	BMP-7 modulates ovarian steroidogenesis in human GCs [29].
BMP-8	BMP-8 promotes expansion a prevents apoptosis of CCs in vitro [30].
BMP-9	BMP-9 is expressed in the ovary regulating steroidogenesis by GCs [31].
BMP-10	Low BMP-10 expression is correlated with poor progression of ovarian cancer [32].
BMP-11	BMP-11 knockout mice die shortly after birth [33].
BMP-12, BMP-13, BMP-14	No functions have been described in FRS.
BMP-15	A novel BMP-15 mutation has been shown to provide additional understanding of the molecular basis of premature ovarian failure (POF) disease [34].
